# Multi-model forecasts of the ongoing Ebola epidemic in the Democratic Republic of Congo, March–October 2019

**DOI:** 10.1098/rsif.2020.0447

**Published:** 2020-08-26

**Authors:** Kimberlyn Roosa, Amna Tariq, Ping Yan, James M. Hyman, Gerardo Chowell

**Affiliations:** 1 Department of Population Health Sciences, School of Public Health, Georgia State University, Atlanta, GA, USA; 2 Infectious Disease Prevention and Control Branch, Public Health Agency of Canada, Ottawa, Canada; 3 Department of Mathematics, Center for Computational Science, Tulane University, New Orleans, LA, USA; 4 Division of International Epidemiology and Population Studies, Fogarty International Center, National Institute of Health, Bethesda, MD, USA

**Keywords:** real-time forecast, phenomenological models, Ebola, Democratic Republic of the Congo, uncertainty quantification, reporting delay

## Abstract

The 2018–2020 Ebola outbreak in the Democratic Republic of the Congo is the first to occur in an armed conflict zone. The resulting impact on population movement, treatment centres and surveillance has created an unprecedented challenge for real-time epidemic forecasting. Most standard mathematical models cannot capture the observed incidence trajectory when it deviates from a traditional epidemic logistic curve. We fit seven dynamic models of increasing complexity to the incidence data published in the World Health Organization Situation Reports, after adjusting for reporting delays. These models include a simple logistic model, a Richards model, an endemic Richards model, a double logistic growth model, a multi-model approach and two sub-epidemic models. We analyse model fit to the data and compare real-time forecasts throughout the ongoing epidemic across 29 weeks from 11 March to 23 September 2019. We observe that the modest extensions presented allow for capturing a wide range of epidemic behaviour. The multi-model approach yields the most reliable forecasts on average for this application, and the presented extensions improve model flexibility and forecasting accuracy, even in the context of limited epidemiological data.

## 1. Introduction

There is a long, rich history of using mathematical models to study the spread and control of infectious diseases [1–3]. For instance, mathematical models can provide insight on the impact of different transmission mechanisms and interventions [4–6], estimate transmission potential across different pathogens and social settings [7,8] and evaluate optimal strategies for resource allocation [9,10]. Mathematical models can forecast, identify and predict the morbidity and mortality patterns in infectious disease outbreaks in near real time (e.g. [10,11]). Public health officials can use the model short-term projections to inform public health interventions during an outbreak [4,12–18].

Many modelling studies rely on historical epidemic data to evaluate the effectiveness of the model for forecasting an epidemic [5,6,13,15]. By contrast, real-time studies aim to generate predictions as the epidemic unfolds [4,7,10–12,19–21]. These real-time studies present with additional challenges, as surveillance data are often affected by underreporting, misclassification and reporting delays [21,22]. Fortunately, standard statistical methods can be useful to adjust short-term incidence trends for reporting delays and ‘nowcast’ data in real time [21,23,24].

The 2018–2020 Ebola epidemic in the Democratic Republic of the Congo (DRC) was initially declared on 1 August 2018. As of 26 April 2020, a total of 3461 cases have been reported, mostly in the provinces of North Kivu and Ituri (with six cases from the province of South Kivu) [25]. The outbreak has now largely been brought under control; however, small resurgences are still being reported over a year and a half after the start of the outbreak. Despite vaccination and other preventative efforts, the outbreak has persisted largely due to long-standing conflict in the region, including recurrent violent attacks targeting Ebola treatment centres and healthcare teams [25–27]. Particularly, regions of North Kivu and Ituri have been destabilized, leading to conflict from more than 70 armed militant groups [28]. In addition to violence, a complicated history of humanitarian intervention has hindered the Ebola response efforts, impacting epidemiological surveillance and contact tracing efforts, including temporary suspension of Ebola response activities [22,26,28,29]. The multiple Ebola resurgences associated with these instabilities have resulted in a multimodal incidence pattern (see electronic supplementary material, figure S1) [7,30]. The complex characteristics and trajectory of this outbreak pose an unprecedented challenge for forecasting the trajectory of the epidemic in real time.

In February 2019, a sharp increase in cases and transmission was observed, coinciding with deteriorating security, targeted attacks on response teams and decreasing trust in the Ebola response efforts [31,32]. Previous studies have provided real-time forecasts at different time points of the 2018–2020 Ebola epidemic in the DRC (electronic supplementary material, figure S1) using various approaches, including a semi-structured model that relies on nowcasting [21], stochastic and auto-regressive models that incorporate historical data [20], as well as a sub-epidemic wave framework [30], which we also use here. While each of these approaches performed well for fitting and forecasting the trajectory of the outbreak in 2018 and early 2019, each model failed to predict the case resurgence observed in February 2019, resulting in forecasts that drastically underestimated the true cumulative case count to date. Therefore, we focus model calibration in this study on the large 2019 resurgence to better project the upcoming epidemic trajectory. This also allows for the implementation of simpler models, including models that only allow for a single peak.

We systematically compare real-time forecasts (one to four weeks ahead) for the ongoing Ebola epidemic in the DRC using seven dynamic models of variable complexity. Our models range from simple scalar differential equation models, such as the standard logistic growth and Richards models, to more complex dynamic models that capture a diversity of epidemic trajectories, such as multimodal outbreaks. These include extensions of the recently developed sub-epidemic wave framework consisting of systems of differential equations [30], an extended Richards model that incorporates an endemic state and a double logistic growth model that supports incidence curves with two peaks [33]. We also present a performance-based multi-model approach that incorporates the four single equation models in order of increasing complexity. We stratify forecasting performance within specific forecasting phases, as defined by the multi-model approach.

## 2. Data and methods

### 2.1. Incidence data of the DRC Ebola epidemic and adjusting for reporting delays

We retrieve weekly case incidence data for the 2018–2020 Ebola epidemic in the DRC from the epidemic curves published weekly in the World Health Organization (WHO) Situation Reports [25]. Many complicating factors, including the recurrent violent attacks and widespread public distrust, have hindered the Ebola surveillance and containment efforts in the DRC [26,34] and resulted in delays in reporting the true incidence curve [22]. Outbreak curves describing epidemic spread in near real time can be distorted by reporting delays, so we adjust the crude incidence for reporting delays using statistical methods.

Reporting delay is defined as the time lag between the time of onset and the time when the case is reported and entered into the database [33]. Reporting delays occur for multiple reasons, including difficulty in tracing and monitoring cases, attacks on health workers and health centres, resistance of sick individuals to seek treatment as soon as symptoms occur, inefficient surveillance systems and population mobility [35]. We use a non-parametric actuaries method that adapts survival analysis for use with right truncated Ebola weekly incidence data by employing point estimation based on reverse time hazards to statistically adjust for reporting delays based on the empirical distribution of the delays [24,36,37]. This allows us to estimate the number of occurred but not yet reported events at a given point in time owing to incomplete case reporting. This well-established method involves expressing the conditional reporting delay distribution as the product of conditional probabilities. The adjusted incidence data are obtained by appropriately dividing the observed number of cases by the reporting delay distribution. The reporting delay adjustment is given by

$$\begin{array}{c} \hat{i}(t)=\;\frac{N(t;C)}{\prod_{u=C-t+1}^{C}[1-\hat{g_{u}}]}=\frac{N(t;C)}{\hat{\text{Pr}}(X\leq C-t|X\leq C)},\;\\ t=\;1,\;2,\ldots ,C. \end{array}$$

In other words, the number of event onsets at time *t* observed by the current time *C*, or *N*(*t*; *C*), is a proportion of the number of onsets occurring at time *t*, *i*(*t*):

N(t;C)=i(t) ∗ Pr(X≤C−t|X≤C).

This proportion is estimated by

$$\hat{Pr}(X\leq C-t|X\leq C)=\prod_{u=C-t+1}^{C}[1-\hat{g_{u}}],$$

where $\hat{Pr}(X\leq C-t|X\leq C)$ represents the proportion of events with a delay X≤C−t, out of those with a delay *X* ≤ *C* [23,28]. Thus $\hat{Pr}(X\leq C-t)$ represents the percentage of events at times *t* reported by time *C*. This method works better when case counts are moderately large, as larger numbers in data provide greater precision and narrower confidence intervals. We sequentially analyse incidence data from consecutive Situation Reports to adjust for reporting delays as more information becomes available.

### 2.2. Model calibration and forecasting approach

We conducted 29 week-to-week forecasts between 11 March and 23 September 2019. Each forecast was fitted to the reporting delay-adjusted weekly incidence from data reported in Situation Reports 33–61, between 19 March 2019 and 1 October 2019. The uncertainty in the reporting delay is greatest in the most recently reported (last observed) weekly incidence data point; thus, we exclude the last weekly incidence data point in the analysis (lag of one week) (electronic supplementary material, figure S9). The first model calibration process relies on five incidence weeks: 11 February–11 March 2019, with the latest snapshot of the epidemic corresponding to Situation Report 33 (19 March 2019). Sequentially, models are re-calibrated each week using the most up-to-date adjusted incidence curve, meaning that the length of the calibration period increases by one week with each new weekly published WHO Situation Report [25].

The set of model parameters, *Θ* = (*θ*_1_, *θ*_2_, …, *θ*_m_) (electronic supplementary material, table S1), is estimated using nonlinear least-squares fitting to minimize the sum of squared errors between the model prediction *f*(*t*, *Θ*) and the data *y_t_*. The estimated parameters $\hat{\Theta }=\text{argmin}\sum_{t=1}^{n}{\left(f\;(t,\Theta )-y_{t}\right)}^{2}\;\text{define}\;\text{the best-fit model}f(t,\hat{\Theta }\;)$. To test the uniqueness of the best-fit model, we initialize the parameters for the nonlinear least-squares method over a wide range of feasible parameters from a uniform distribution using Latin hypercube sampling. Further, we fix the initial condition according to the first data point.

We use a parametric bootstrap approach to quantify parameter uncertainty and estimate prediction intervals (PIs), which involves resampling with replacement of incidence data assuming a Poisson error structure [38]. Our calibration results represent *M =* 300 resampled datasets that are refitted to obtain *M* new parameter estimates. Model fits are used to obtain 95% confidence intervals for each parameter [38].

Each of the *M* model fits is used to generate *m* = 30 simulated data curves with Poisson noise; these 9000 (*M x m*) curves are then used to construct the 95% PIs for the forecasting period of one to four weeks (*h* = 1, 2, 3, 4). We give a detailed description of this parameter estimation method in prior studies [38–40].

### 2.3. Performance metrics

We used the following model performance metrics to assess the quality of the model fit and forecasting performance (*h =* 1–4 weeks ahead). For calibration performance, we compare model fit with the adjusted observed data, whereas we compare forecasts with the raw incidence data reported four weeks ahead of the last date of the calibration period.

The mean squared error (MSE) and the mean absolute error (MAE) assess average deviations of the model to the observed data

$$\text{MSE}=\;\frac{1}{n}\overset{n}{\underset{i=1}{\sum }}{\left(Y_{i}-{\hat{Y}}_{i}\right)}^{2}$$

and

$$\text{MAE}=\;\frac{1}{n}\overset{n}{\underset{i=1}{\sum }}|Y_{i}-{\hat{Y}}_{i}|,$$

where *Y_i_* is the data, ${\hat{Y}}_{i}$ is the model prediction and *n* is the number of data points in the interval. For the calibration period, *n* equals the number of data points calibrated to, and for the forecasting period, *n = h =* 1, 2, 3, 4 for 1–4 weeks ahead forecasts, respectively.

To assess model uncertainty and performance of PIs, we use PI coverage and mean interval score (MIS) [41]. PI coverage is the fraction of data points that fall within the 95% PI, calculated as

$$\text{PI coverage}=\frac{1}{n}\overset{n}{\underset{t=1}{\sum }}1\{Y_{t}>L_{t}\;\cap \;Y_{t}<U_{t}\},$$

where *n* is the length of the period, *L_t_* and *U_t_* are the lower and upper bounds of the 95% PIs, respectively, *Y_t_* are the data and **1** is an indicator variable that equals 1 if *Y_t_* is in the specified interval and 0 otherwise.

The mean interval score considers the width of the interval as well as the coverage, with a penalty for data points not included within the PIs. The MIS is calculated as

$$\text{MIS}=\;\frac{1}{n}\overset{n}{\underset{t=1}{\sum }}(U_{t}-L_{t})+\frac{2}{\alpha }(L_{t}-Y_{t})1\{Y_{t}<L_{t}\}+\frac{2}{\alpha }(Y_{t}-U_{t})1\{Y_{t}>U_{t}\},$$

where *n* is the length of the period, *L_t_* and *U_t_* are the lower and upper bounds of the 95% PIs, respectively, *Y_t_* are the data, *α* is the significance level (*α* = 0.05) and **1** is an indicator variable that equals 1 if *Y_t_* is in the specified interval and 0 otherwise [41]. Therefore, if the PI coverage is 1, the MIS is the average width of the interval across each time point. For two models with equivalent PI coverage, a lower MIS indicates narrower intervals.

### 2.4. Forecasting strategy

We evaluate short-term forecasts in real time using seven dynamic models: four single-equation models of increasing complexity, a multi-model approach and two sub-epidemic wave models whose complexity depends on the temporal pattern of the epidemic. Features such as number of parameters, number of equations and ability to capture varying dynamics are provided in table 1. A brief overview of the models is provided below, and the electronic supplementary material contains additional details to fully define the models.

**Table 1.** Structural characteristics of the seven dynamic models used for real-time forecasting. The multi-model approach encompasses 1–4 of the single-equation models in the first four rows based on prediction interval coverage of the individual models (following the schematic in figure 1). For the two sub-epidemic models, the number of differential equations is equal to the number of sub-epidemics estimated by the model (*n*).

	no. of parameters estimated	no. of differential equations	supports endemic state	supports two peaks	supports oscillations
logistic	2	1	no	no	no
Richards	3	1	no	no	no
endemic Richards	5	1	yes	no	no
double logistic	6	1	yes	yes	no
multi-model	2–6	1	yes	yes	no
sub-epidemic I	5	≥1 (*n*)	yes	yes	yes
sub-epidemic II	5	≥1 (*n*)	yes	yes	yes

The two-parameter logistic growth model is useful as a simple benchmark for comparing the performance of the more complex models. The well-known three-parameter Richards model extends the logistic growth model to include an additional parameter to allow for asymmetry in the decline of the epidemic curve [42,43]. If the data follow a symmetric logistic trajectory, then the logistic model can accurately fit the data. However, if the incidence is asymmetric, then the Richards model will yield a better fit.

We also introduce and apply an extension of the Richards model that consists of five parameters and an endemic state; therefore, we denote this as the ‘endemic Richards’ model [33]. If the epidemic declines to a steady state or endemic level, rather than declining to extinction, the Richards model will under-predict the future incidence. When this happens, short-term forecasts derived using the endemic Richards model tend to outperform the simple Richards model.

The model is then extended to a six-parameter ‘double logistic’ model that supports trajectories with double peaks or temporary steady states followed by a secondary decline (electronic supplementary material, figure S2) [33]. When data points fall outside the PI coverage of the endemic state assumed by the endemic Richards model, a decline greater than the assumed level of statistical noise is indicated; this means that it is likely to be a true decline, rather than stochasticity. Therefore, the endemic Richards model will overpredict the incidence, and the double logistic model will be more appropriate.

**Figure 1.**
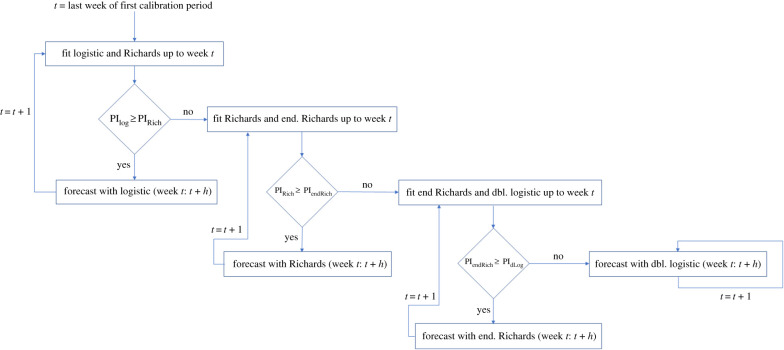
Schematic for the performance-based multi-model approach. The flow diagram describes the process of sequentially choosing models based on prediction interval (PI) coverage to provide forecasts for each weekly projection, following the order: logistic, Richards, endemic Richards and double logistic. Here, *h* is the length of the forecasting period.

We introduce a multi-model approach (see next section) that sequentially uses the four single-equation models mentioned above. For this purpose, we compare the models in order of increasing complexity (table 1) and assess PI coverage to determine which model to employ for the forecast. Our multi-model algorithm is summarized in figure 1.

The most flexible model we use is a sub-epidemic wave model that supports complex temporal dynamic patterns, such as oscillating dynamics leading to endemic states or damped oscillations [30]. We incorporate two variations of sub-epidemic decline: exponential decline, as presented in [30], and a new extension with an inverse decline function; each of the variations includes five fitting parameters. This approach assumes that multiple underlying sub-epidemics shape the aggregate reported epidemic curve, where each sub-epidemic is modelled using a generalized logistic growth model. These combine to create an epidemic wave composed of *n* overlapping sub-epidemics modelled using a system of *n* coupled differential equations.

### 2.5. Multi-model approach

For the multi-model approach, we compare the four single-equation models in order of increasing complexity (table 1), and we assess the PI coverage of the calibration period to determine when/if to switch models, as summarized in our schematic shown in figure 1.

We begin at the initial forecasting week by comparing the calibration PI coverage between the logistic and Richards models. When the calibration PI coverage of the logistic model is greater than or equal to the PI coverage of the Richards model, we provide forecasts with the logistic model. When the PI coverage of the Richards model is greater, we then switch to comparing the Richards model with the endemic Richards, and the iterative process continues as such (figure 1).

We define the *forecasting phases* as the time intervals corresponding to the Situation Reports for which each model is used. That is, each time the method switches to a new forecasting model, a new forecasting phase is initiated, and there will be as many forecasting phases as models used. Notably, any number of the four models could make up the multi-model approach. For example, if the Richards model provides higher PI coverage than the logistic model at time *t*_switch_ and the endemic Richards model has higher PI coverage than the Richards model at time *t*_switch_, then the Richards model would not be used for any forecasts (figure 1). The models are analysed in the explicit order reported, and once a model is switched to, there is no switching back to simpler models.

## 3. Results

We compare the calibration and real-time short-term forecasting performance of the seven models in table 1 on the major Ebola resurgence between 11 March 2019 and 23 September 2019. We further assess performance within each forecasting phase, as defined by the multi-model approach. The electronic supplementary material contains additional figures of the model fits (electronic supplementary material, figures S3–S8).

### 3.1. Forecasting phases

**Figure 2.**
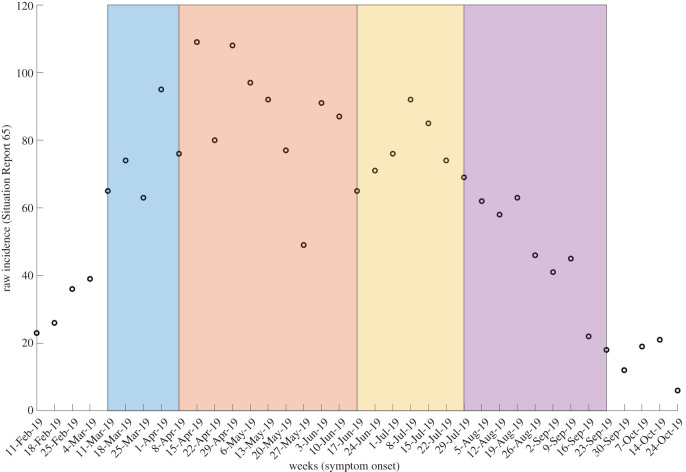
Visual representation of the forecasting phases defined by the multi-model approach. Raw data from Situation Report 65 are shown with the periods for which each model was used: logistic growth model (blue), Richards model (red), endemic Richards (yellow) and double logistic (purple).

**Figure 3.**
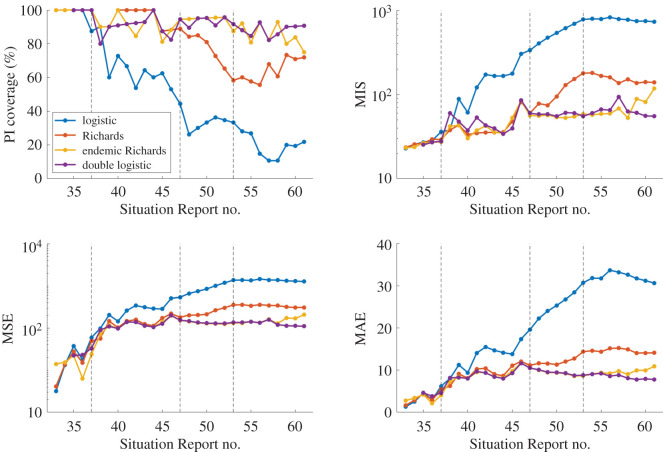
Calibration period metrics for each Situation Report for the four individual models included in the performance-based multi-model approach: logistic growth model (blue), Richards model (red), endemic Richards (yellow) and double logistic (purple). Higher prediction interval (PI) coverage and lower mean interval score (MIS), mean squared error (MSE) and mean absolute error (MAE) indicate better performance. PI coverage is used to indicate the ‘switch’ in models, as designated by the vertical dashed lines.

As explained in the methods, we define our *forecasting phases* by assessing the calibration PI coverage of the four single-equation models as defined by the multi-model approach (electronic supplementary material, figure S3). The following forecasting phases were obtained: weekly forecasts with the logistic model for 11 March–1 April 2019 (data from Situation Reports 33–36), with the Richards model for 8 April–10 June 2019 (Situation Reports 37–46), with the endemic Richards model for 17 June–22 July 2019 (Situation Reports 47–52) and with the double logistic model for 29 July–23 September 2019 (Situation Reports 53–61) (figure 2). We will refer to these consecutive forecasting phases as: incline, oscillating I, oscillating II and decline, respectively. These break points based on PI coverage are also consistent with the timing of where the models begin to deviate with respect to each of the other calibration performance metrics: MSE, MAE and MIS (figure 3).

In general, the resulting forecasting phases obtained by our multi-model approach are consistent with our rationale for incorporating the dynamic models supporting different dynamics. Data from 11 February–1 April represent the early growth dynamics of the 2019 resurgence; thus, we define the first phase (11 March–1 April) as the incline phase, for which the simple logistic model is sufficient for fitting the data (electronic supplementary material, figure S3).

In the next phase beginning 8 April, oscillating I, the outbreak begins to fluctuate, and the Richards model outperforms the logistic model. As new observations are added to the weekly incidence curve, the deviations between the logistic model and data become more pronounced (figure 3 and electronic supplementary material, figure S3). The trajectory continues on a sustained oscillating pattern through the next phase, oscillating II, so the endemic Richards model provides a better model fit than the simple Richards model.

On 29 July, the switch to the final model and the decline phase is initiated (figure 3). From 29 July to 2 September (Situation Reports 53–58), the endemic Richards and double logistic model perform comparably in each of the calibration metrics. However, the double logistic model outperforms the endemic Richards model between 9 September and 23 September (Situation Reports 59–61). This is the point where the trajectory falls outside the 95% PI obtained using the endemic Richards model, suggesting a need for a model that can capture the declining trend (electronic supplementary material, figures S6 and S7). The double logistic model outperforms the other single-equation models in capturing the full national incidence pattern up to 23 September (table 2 and figure 3).

**Table 2.** Calibration performance of the seven dynamic models calibrated to the data through the last Situation Report (SR) of each time interval. The time intervals are defined by the multi-model approach, where the switch in model/interval is driven by the prediction interval coverage of the model used. The multi-model results are equivalent to the individual model used for that time interval (logistic, Richards, endemic Richards and double logistic, respectively).

	PI coverage (%)	MIS	MSE	MAE
1 April 2019 (SR 36)				
*logistic*	**100.0^a^**	28.4	17.8	3.0
*Richards*	**100.0^a^**	29.4	15.2	2.9
*endemic Richards*	**100.0^a^**	27.2	**6.4^a^**	**2.1^a^**
*double logistic*	**100.0^a^**	**27.1^a^**	23.2	3.8
*multi-model*	**100.0^a^**	28.4	17.8	3.0
*sub-epidemic I*	**100.0^a^**	28.6	13.9	2.7
*sub-epidemic II*	**100.0^a^**	28.7	13.9	2.9
10 June 2019 (SR 46)				
*logistic*	52.9	302.9	511.2	17.4
*Richards*	88.2	81.9	220.0	12.1
*endemic Richards*	88.2	84.2	**199.9^a^**	11.7
*double logistic*	82.4	85.7	200.7	11.7
*multi-model*	88.2	81.9	220.0	12.1
*sub-epidemic I*	82.4	74.7	213.8	**11.1^a^**
*sub-epidemic II*	**94.1^a^**	**70.9^a^**	213.2	11.4
22 July 2019 (SR 52)				
*logistic*	34.8	696.0	1191.8	28.5
*Richards*	65.2	153.3	306.7	12.7
*endemic Richards*	**95.7^a^**	54.6	**124.9^a^**	**8.6^a^**
*double logistic*	**95.7^a^**	60.3	129.0	8.7
*multi-model*	**95.7^a^**	54.6	**124.9^a^**	**8.6^a^**
*sub-epidemic I*	87.0	**49.7^a^**	129.8	9.4
*sub-epidemic II*	87.0	**49.7^a^**	129.9	9.4
23 September 2019 (SR 61)				
*logistic*	21.9	732.78	1281.7	30.6
*Richards*	71.9	139.1	312.0	14.1
*endemic Richards*	75.0	117.9	210.7	10.9
*double logistic*	**90.6^a^**	55.3	**110.9^a^**	7.8
*multi-model*	**90.6^a^**	55.3	**110.9^a^**	7.8
*sub-epidemic I*	**90.6^a^**	**52.7^a^**	126.6	8.9
*sub-epidemic II*	**90.6^a^**	61.5	122.2	**7.5^a^**

^a^Best performance with regards to the performance metric (column), i.e. highest prediction interval (PI) coverage and lowest mean interval score (MIS), mean squared error (MSE) and mean absolute error (MAE).

### 3.2. Calibration performance

The calibration performance metrics across phases, based on the last date of calibration within each of the four phases, are given in table 2. For data through the incline phase, each of the models provides 100% PI coverage and very similar MIS, with the double logistic having the lowest (MIS = 27.1), followed by the endemic Richards (MIS = 27.2). The endemic Richards model has significantly lower MSE and MAE for the incline phase than the other models (table 2).

For data through oscillating I, the sub-epidemic model type II has the highest PI coverage (94.1%) and lowest MIS (70.9), while the endemic Richards and sub-epidemic type I have the lowest MSE and MAE, respectively (table 2). For data through oscillating II, the endemic Richards, multi-model and the double logistic model have the highest PI coverage (95.7%), while sub-epidemic types I and II have the lowest MIS (49.7). The endemic Richards, which corresponds with the multi-model approach for oscillating II, also has the lowest MSE and MAE.

When fitting all the available data through 23 September, or the decline phase, the double logistic, multi-model and both sub-epidemic models perform best in terms of PI coverage (90.6%); however, the other metrics are split between these models (table 2). The three simplest models perform poorly on the full data, supporting the need for more flexible models to capture the complex dynamics of the epidemic.

**Figure 4.**
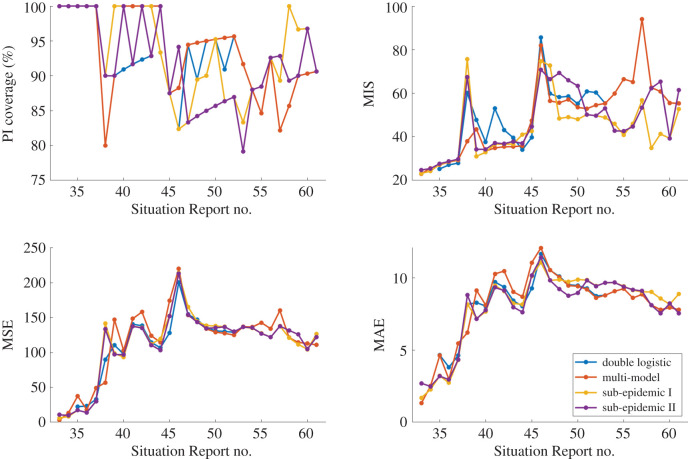
Calibration period metrics for each Situation Report for the double logistic model (blue), multi-model approach (red) and sub-epidemic model types I (yellow) and II (purple). Higher prediction interval (PI) coverage and lower mean interval score (MIS), mean squared error (MSE) and mean absolute error (MAE) indicate better performance. Further, the multi-model approach overlaps the double logistic model for Situation Reports 53–61.

**Figure 5.**
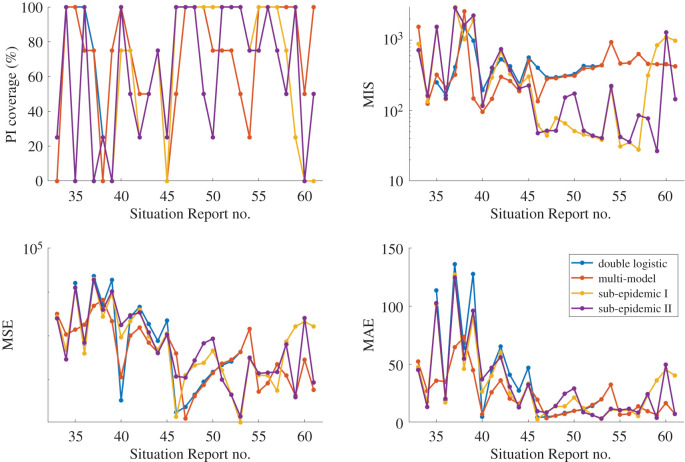
Forecasting period metrics (forecasting length *h* = 4 weeks) for each Situation Report for the double logistic model (blue), multi-model approach (red), and sub-epidemic model types I (yellow) and II (purple). Higher forecasting prediction interval (PI) coverage and lower mean interval score (MIS), mean squared error (MSE) and mean absolute error (MAE) indicate better forecasting performance. Again, the multi-model approach overlaps the double logistic model for Situation Reports 53–61.

Weekly calibration performance across the entire incidence curve using the double logistic model, the performance-based multi-model approach and the two sub-epidemic models is displayed in figures 4 and 5. Goodness-of-fit metrics do not point to a single winner or ‘best’ model (figure 4). In terms of mean model fit and error, the models perform comparably with regards to MSE and MAE. The models show variation in PI coverage and MIS; however, the curves repeatedly overlap, suggesting that there is not necessarily a clear best model across the full epidemic trajectory.

### 3.3. Forecasting performance

The forecasting results by phase are presented in table 3. For the incline phase, the endemic Richards model provides forecasts with substantially lower MSE and MAE than any other model. The double logistic model has the highest PI coverage (100%) and lowest MIS (207.3); however, the MSE is more than 14 times higher than that of the endemic Richards (table 3). Thus, the high coverage can be attributed to very wide prediction intervals (electronic supplementary material, figure S6).

**Table 3.** Average forecasting performance of four-week ahead forecasts across each Situation Report within the four distinct forecasting phases. The time intervals are defined by the multi-model calibration results (corresponding with figure 2), where the switch in model/interval is driven by the prediction interval coverage of the model used; therefore, the multi-model results are equivalent to the individual model used for their respective time intervals.

	PI coverage (%)	MIS	MSE	MAE
Situation Reports 33–36: incline				
*logistic*	68.8	553.5	1857.5	37.5
*Richards*	81.3	428.5	2072.1	36.1
*endemic Richards*	81.3	247.9	**580.0^a^**	**18.6^a^**
*double logistic***^b^**	**100.0^a^**	**207.3^a^**	8308.6	66.4
*multi-model*	68.8	553.5	1857.5	37.5
*sub-epidemic I*	56.3	672.3	3987.3	46.0
*sub-epidemic II*	56.3	646.1	4050.6	45.4
Situation Reports 37–46: oscillating I				
*logistic*	37.5	859.2	1951.3	36.7
*Richards*	55.0	**468.7^a^**	**1883.0^a^**	**33.9^a^**
*endemic Richards*	**65.0^a^**	494.4	4613.2	51.1
*double logistic*	55.0	559.9	5922.6	56.2
*multi-model*	55.0	**468.7^a^**	**1883.0^a^**	**33.9^a^**
*sub-epidemic I*	42.5	794.3	4123.0	46.6
*sub-epidemic II*	45.0	889.1	4424.7	50.1
Situation Reports 47–52: oscillating II				
*logistic*	0.0	2192.5	4039.0	62.9
*Richards*	0.0	684.4	1081.4	31.7
*endemic Richards*	87.5	71.3	131.6	**8.7^a^**
*double logistic*	87.5	68.7	**129.8^a^**	8.8
*multi-model*	87.5	71.3	131.6	**8.7^a^**
*sub-epidemic I*	**100.0^a^**	**54.5^a^**	208.1	12.6
*sub-epidemic II*	79.2	88.1	344.9	15.1
Situation Reports 53–61: decline				
*logistic*	8.3	837.8	1026.4	27.8
*Richards*	55.6	**115.1^a^**	**183.7^a^**	**11.9^a^**
*endemic Richards*	16.7	784.1	1482.4	36.1
*double logistic*	**80.6^a^**	118.5	304.5	13.2
*multi-model*	**80.6^a^**	118.5	304.5	13.2
*sub-epidemic I*	63.9	401.4	747.0	20.4
*sub-epidemic II*	69.4	219.5	453.7	14.4

^a^Best performance with regards to the performance metric (column), i.e. highest prediction interval (PI) coverage and lowest mean interval score (MIS), mean squared error (MSE) and mean absolute error (MAE).

^b^The double logistic model averages are for Situation Reports 35 and 36.

For oscillating I, the endemic Richards model provides the highest PI coverage of future data points (65.0%), while the Richards model has the lowest MSE, MAE and MIS, which correlates with the multi-model approach having the lowest error and MIS as well (table 3).

As more complicated dynamics emerge, the simpler models fail to predict the epidemic trajectory accurately. For oscillating II, both the logistic and Richards models have PI coverage of 0% with high error (table 3). The sub-epidemic model type I outperforms all other models on PI coverage (100%) and MIS (54.5) for this phase, indicating it has high PI coverage without significantly higher error. The endemic Richards, double logistic and multi-model approach yield the lowest MAE and MSE for oscillating II.

For the final phase, the decline phase, the double logistic and multi-model approach yield the highest forecast PI coverage (80.6%). Interestingly, the simple Richards model provides forecasts with the lowest MIS, MSE and MAE for the last phase; however, PI coverage is only 55.6% (table 4). Further, if we had continued conducting forecasts past the end of the study period, the Richards model would have failed to capture the continued endemic state observed. The double logistic and multi-model approach rank second in MIS, MSE and MAE, so while there is not a clear best model, the double logistic and multi-model approach highly perform across all of the metrics.

## 4. Discussion

We conducted a systematic comparison of seven models for short-term real-time forecasting of the ongoing 2018–2020 Ebola outbreak in the DRC. A well-defined performance-based approach was used to identify distinct epidemic phases for which to employ different models to capture the complex trajectory of the epidemic. By using different models for different phases of an epidemic, the approach can account for significant changes in transmission dynamics over the course of the outbreak, ranging from a simple logistic curve to incidence curves with oscillatory behaviour, as observed in the DRC (figure 3).

The first defined phase, incline, covers the sharp increase in cases observed in late February–early March, which followed an increase in armed attacks, including the burning of Ebola treatment centres in Katwa and Butembo [31]. Specifically, February 2019 recorded the highest monthly incidence of armed attacks, corresponding with the increase in cases observed in the incline phase. The following two phases represent oscillating dynamics, which correspond with continued violent attacks and increasing community resistance that deterred response activities [28,31]. As the incidence of violent attacks decreased in July 2019, cases levelled out and eventually showed a substantial decline for the final decline phase.

The double logistic model and the sub-epidemic models (types I and II) provide the best fit to the incidence trajectory through the study period (table 2); however, in general, goodness of fit was not found to be correlated with forecasting performance. While the sub-epidemic models often provide the best fit to the calibration data (table 2), they were less successful in forecasting short-term dynamics of the epidemic (table 3). We observed that the sub-epidemic forecasts in the decline phase perform poorly, as the trajectory is declining while the models are predicting another upturn in cases or sub-epidemic waves (electronic supplementary material, figures S7 and S8). The sub-epidemic model, with an inverse decline function (type II), is more successful at capturing the future declining trajectory in Situation Reports 58–61, whereas the version with the exponential decline (type I) cannot predict the declining trend observed in the following weeks (table 3; electronic supplementary material, figures S7 and S8).

The multi-model approach provides the most consistent forecasts, in terms of average MSE and MAE, throughout our study period (electronic supplementary material, table S2). Even when broken into phases, the multi-model approach performs best in at least one of the forecasting metrics for each forecasting phase, which was not the case for any other model (table 3). This general multi-model approach can be adapted to other epidemic scenarios, such as epidemics of emerging pathogens or those occurring in regions with unstable sociopolitical climate, as the models are phenomenological and do not require biological information or knowledge of specific disease transmission processes. However, the four models incorporated here may not be appropriate for all outbreak scenarios. For example, these models do not allow for a higher second peak. This approach would also have failed to predict the February 2019 resurgence, like the other early projections.

The general multi-model approach can be adapted to incorporate any sequence of models. For disease outbreaks with more epidemiological data, specific disease mechanisms can be incorporated in compartmental models that increase in complexity as more outbreak characteristics are elucidated. As model complexity increases, however, the uncertainty of model estimates must be considered. Here, the models build upon each other and have very similar estimates for the early phase, so we rely on PI coverage as our ‘switch’ metric to remain at a simpler model while they all have equivalent coverage. This could potentially be problematic for more complex models, as very wide intervals, such as (0, inf.), would perform ‘better’ in terms of PI coverage, leading to high uncertainty in forecasts. In this situation, one may consider MIS to classify the phases, rather than PI coverage.

Another modelling approach rapidly gaining traction in epidemiological literature is ensemble modelling, which involves incorporating multiple models in a complementary manner [44–46]. Rather than a sequential multi-model approach, future work could rely on an ensemble modelling approach based on a combination of simple dynamic models. With an ensemble approach, we would have the option to base the contribution of each model on calibration performance, rather than choose one model based on calibration as we did here. Another option is to weight the models based on the forecasting performance of prior weeks; however, in this study, forecasting performance in one phase is not clearly predictive of performance in the following phase (table 3). The use of an algorithm like that presented here could supplement ensemble models to define distinct epidemic phases, which may yield better projections than separating data by standard intervals.

Reviews of real-time forecasting throughout the historic 2014–2015 Ebola epidemic found that forecasting uncertainty is higher in the beginning stages of an outbreak and decreases over time [16,17]; however, this was not observed in the 2018–2020 Ebola epidemic. Fluctuations in error and MIS do not reveal a declining pattern in forecast uncertainty as the epidemic progresses. This highlights the challenge of forecasting the complicated dynamics of this epidemic, where increasing the amount of available data does not necessarily decrease the uncertainty of estimates.

The unpredictable social components of the epidemic on the ground in the DRC are major limitations to the study. While we adjust the reported data in real time by the week of symptom onset, we do not know the true stable incidence pattern when forecasts are generated. Further, we rely on phenomenological models, which are particularly valuable for providing rapid predictions of epidemic trajectory in complex scenarios; however, they do not incorporate disease-specific mechanisms or explicitly account for interventions. Because the models do not explicitly account for behaviour changes or interventions, projections from the models should be assessed with caution and are only suitable for short-term predictions. The sub-epidemic modelling framework is the most flexible presented here, and only the two sub-epidemic models are able to predict a resurgence or second wave; however, the variations of the model applied here can only predict constant or decreasing sub-epidemic wave sizes, and thus cannot accommodate larger subsequent waves, as observed in the DRC outbreak. Therefore, none of the models employed here would have anticipated the 2019 disease resurgence, but, when applied from the start of the resurgence, they can be used to forecast the following trajectory in real time.

In conclusion, while the forecasting models introduced here are relatively simple, we are encouraged by the short-term forecasting performance of the model extensions, especially when applied to such a complex, non-traditional epidemic trajectory. While longer term predictions may not be feasible, short-term predictions like those presented assist public health workers with decisions regarding targeted interventions, resource allocation, and preparation for healthcare settings, laboratories and more. This work suggests that a multi-model framework, such as the one presented here, can identify distinct forecasting phases that allow the model to adjust for changing dynamics. Further, the general approach is flexible and can be adapted to many different model combinations and outbreak scenarios. Forecasting challenges during the DRC outbreak underscore the need for more research into flexible real-time forecasting approaches, especially when the dynamics exhibit complex temporal patterns.

## Supplementary Material

Supplementary Material

## Data accessibility

Data and code for reporting delay adjustment and forecasting are available as part of the electronic supplementary material.
