# Supplementary material for: Multi-model forecasts of the ongoing Ebola epidemic in the Democratic Republic of Congo, March–October 2019
Source: J R Soc Interface. 2020 Aug 26;17(169):20200447. doi: 10.1098/rsif.2020.0447 (PMC7482568; doi:10.1098/rsif.2020.0447)
Supplement: Supplementary Material [file rsif20200447supp1.docx]

**Supplemental material for: Multi-model forecasts of the ongoing Ebola epidemic in the Democratic Republic of Congo, March** – **October 2019**

Kimberlyn Roosa, Amna Tariq, Ping Yan, James M. Hyman, & Gerardo Chowell

**Model descriptions:**

*Logistic growth model*

This 2-parameter model is useful as a simple benchmark to compare the performance of more elaborate models. The logistic growth model extends the exponential growth model to include a saturation phase that culminates in a final epidemic size, or maximum carrying capacity, *K*. The logistic model is as follows

$$\dot{C}\left( t \right)=rC\left( t \right)\left( 1-\frac{C\left( t \right)}{K} \right),$$

where *r* is the growth rate, and *K* is the final epidemic size. It is worth mentioning that this model was one of the models included in the Ebola forecasting challenge inspired by the 2014-15 Ebola epidemic [1, 2]. The logistic model generates symmetric bell-shaped incidence curves; therefore, it will tend to perform worse as the outbreak pattern deviates from a symmetric trajectory.

*Richards model*

The well-known Richards model is a simple extension of the logistic growth model that adds one more scaling parameter θ (3 parameters total) to allow for deviation from symmetric S-shaped dynamics [3, 4]*.* This simple yet flexible model has been applied to fit cholera dynamics [5], estimate sub-critical transmissibility of Zika virus [6], and also to the Ebola forecasting challenge [1, 2]. The Richards model is as follows

$\dot{C}\left( t \right)=rC(t)\left( 1-{(\frac{C\left( t \right)}{K})}^{\theta} \right)$,

where θ determines the deviation from symmetry, and again *r* is the growth rate, and *K* is the final epidemic size.

*Endemic Richards model*

Simple growth models such as the logistic and Richards models frequently support incidence trajectories that monotonically decline to 0 cases after incidence reaches a peak. In contrast, the Ebola incidence pattern observed in DRC after the first case surge in 2018 (Figure S1) suggested the need for a flexible model allowing for a leveling off, or an endemic state. Here, we introduce and apply an extension of the Richards model, with 5 parameters, that incorporates an endemic state as follows:

$$i\left( t \right)=\frac{1}{1+\theta e^{-r\left( t-\alpha\right)}}\left( \frac{ke^{-r\left( t-\alpha\right)}}{1+\theta e^{-r\left( t-\alpha\right)}}+c \right)$$

where $i(t)$ is the case incidence at time *t,* ${lim}_{t\to\infty}i(t) = c$*,* and θ > 0 is a shape parameter that controls the symmetry of $i(t)$, such that the peak incidence occurs at time *t = α* and is given by $i\left( \alpha\right)=\frac{k}{{(1+\theta)}^{1+\frac{1}{\theta}}}+\frac{c}{1+\theta}$ [7]. We refer to this model as the endemic Richards model, as it extends the simple Richards model to include an endemic state, *c*. It is worth noting that this extension reduces to the Richards model when *c = 0* and simplifies to the logistic model when *c = 0* and θ = 1. This model can be utilized for short-term forecasts when the epidemic appears to reach a plateau, or steady state. Estimates of *c* > 0 indicate the epidemic is leveling off to this value, rather than declining to extinction.

Real-time weekly forecasts (4 weeks ahead) with the endemic Richards model from July 29 – August 26, 2019 are published at https://publichealth.gsu.edu/research/ebola-forecast-2/.

*Double logistic model*

To allow the possibility of an outbreak having a secondary decline phase following an initial plateau, we further extend the model into a “double logistic” model with 6 parameters. The double logistic equation is as follows:

$$i\left( t \right)=\frac{k_{1}e^{-r\left( t-\alpha_{1} \right)}}{\left( 1+e^{-r\left( t-\alpha_{1} \right)} \right)^{2}}+\frac{1}{1+e^{-r\left( t-\alpha_{2} \right)}}\left( \frac{k_{2}e^{-r\left( t-\alpha_{2} \right)}}{1+e^{-r\left( t-\alpha_{2} \right)}}+c \right)$$

where *r* is the early growth rate, ${lim}_{t\to\infty}i\left( t \right)= c,$ *k_1_* and *k_2_* are related to the sizes of the peaks, and α_1_ and α_2_ are related to the timing of the respective first and second ‘peaks’ [7]. The double logistic model allows for incidence trajectories with a peak, followed by a decline phase, and then a plateau, followed by another decline phase, before reaching the endemic level *c*; representative fits with varying parameter values are provided (Figure S2).

Real-time weekly forecasts (4 weeks ahead) with the double logistic model from September 2 - October 14, 2019 are published at https://publichealth.gsu.edu/research/ebola-forecast-2/.

*Sub-epidemic wave model*

We have recently developed a sub-epidemic wave model that outperforms short-term forecasts generated using simple models [8]. In particular, this modeling approach supports complex temporal dynamic patterns, such as oscillating dynamics leading to endemic states or damped oscillations. This approach is based on the premise that multiple underlying sub-epidemics shape the aggregate reported epidemic curve. Each underlying sub-epidemic is modeled using a generalized logistic growth model (GLM). The GLM is an extension of the logistic growth model that allows for sub-exponential growth through the ‘scaling of growth’ parameter *p.* The GLM is as follows:

 $\dot{C}\left( t \right)=r{C(t)}^{p}\left( 1-\frac{C\left( t \right)}{K} \right)$,

where *r* is the growth rate, *K* is the final epidemic size, *p =* 1 represents exponential growth, *p* = 0 represents constant growth, and 0 < *p <* 1 represents sub-exponential growth.

We model an epidemic wave composed of *n* overlapping sub-epidemics using a system of coupled differential equations, as follows:

$\dot{C_{i}}\left( t \right)=rA_{i-1}(t){C_{i}(t)}^{p}\left( 1-\frac{C_{i}\left( t \right)}{K_{i}} \right)$,

where *C_i_(t)* is the cumulative number of infections for sub-epidemic *i*, and *K_i_* is the size of the *i*^th^ sub-epidemic (*i* = 1, …, *n*) [8]. Note that the *r* and *p* parameters are the same across sub-epidemics. Therefore, when *n* = 1 and *p* = 1, the sub-epidemic model returns to the simple logistic model. To model the timing of onset for each subsequent wave, we use a *regular* structure, such that the (*i*+1)^th^ sub-epidemic begins when *C_i_(t)* exceeds the threshold *C_thr_*. Further, the sub-epidemics are overlapping, as the (*i*+1)^th^ sub-epidemic begins before the *i*^th^ sub-epidemic completes its trajectory. Thus, we use an indicator variable *A_i_(t),* such that

$$A_{i}\left( t \right)=\left\{ \begin{aligned} 1 C_{i}\left( t \right)>C_{thr} \\ 0 Otherwise \end{aligned} \right., i=1, 2, \ldots, n$$

where 1 ≤ *C_thr_* < *K_0_,* and *A_1_(t)* = 1 for sub-epidemic 1. The size of subsequent sub-epidemics (*K_i_*) can be modeled such that they remain steady or experience a decline. Here, we use two different types of decline to model the size of consecutive sub-epidemics.

*- Type I: Exponential decline of sub-epidemics*

For Type I, we assume the size of each consecutive sub-epidemic declines exponentially. Thus,

$K_{i}=K_{0}e^{-q(i-1)}$,

where *K_0_* is the size of the first sub-epidemic (*K_1_ = K_0_*), and q is the rate of decline of consecutive sub-epidemics. When *q* = 0, the sub-epidemics are of equal size (no decline), and when *q* > 0, the total number of sub-epidemics (*n_tot_*) is finite and depends on *C_thr_, q,* and *K_0_*, as the (*i* + 1)^th^ sub-epidemic is triggered only if *C_thr_ ≤ K_i_.*

$$n_{tot}=\left\lfloor-\frac{1}{q}\left. \ln\left( \frac{C_{thr}}{K_{0}} \right)+1 \right\rfloor\right.$$

Therefore, the total size of the epidemic with *n_tot_* overlapping sub-epidemics is given by:

$$K_{tot}=\sum_{i=1}^{n_{tot}} K_{0}e^{-q(i-1)}=\frac{K_{0}(1-e^{-qn_{tot}})}{1-e^{-q}}$$

Real-time weekly forecasts (4 weeks ahead) with the sub-epidemic model with exponential decline from July 22 – October 14, 2019 are published at https://publichealth.gsu.edu/research/ebola-forecast-1/.

*– Type II: Inverse decline of sub-epidemics*

A new extension of the sub-epidemic wave model incorporates an inverse decline function that characterizes the size of consecutive sub-epidemic sizes, which is given by:

$K_{i}=K_{0}{(\frac{1}{i})}^{q}$.

Again, *q* = 0 represents equal sub-epidemic sizes, or no decline. When *q* > 0, *n_tot_* is finite and given by

$n_{tot}=\left\lfloor\left. \left( \frac{C_{thr}}{K_{0}} \right)^{-\frac{1}{q}} \right\rfloor\right.$;

further, the corresponding total size of the epidemic is given by

$K_{tot}=\sum_{i=1}^{n_{tot}} K_{0}{(\frac{1}{i})}^{q}$.

**Table S1.** Parameter descriptions for estimated parameters of the dynamic models utilized for real-time forecasting. The multi-model approach is excluded, as it encompasses the four single-equation models in the first four rows (Figure S3).

|  | Estimated parameter descriptions |
| --- | --- |
| Logistic | *r –* Growth rate  *K* – Final epidemic size |
| Richards | *r –* Growth rate  *K* – Final epidemic size  *θ –* Deviation from symmetry |
| Endemic Richards | *r –* Growth rate  *k* – Related to final epidemic size  *θ –* Deviation from symmetry  *α –* Timing of peak incidence: $i\left( \alpha\right)=\frac{k}{{(1+\theta)}^{1+\frac{1}{\theta}}}+\frac{c}{1+\theta}$  *c* – Endemic state: ${lim}_{t\to\infty}i(t) = c$ |
| Double logistic | *r –* Growth rate  *k_1_* – Related to size of first peak  *k_2_* – Related to size of second peak  *α_1_ –* Related to timing of first peak  *α_2_ –* Related to timing of second peak  *c* – Endemic state: ${lim}_{t\to\infty}i(t) = c$ |
| Sub-epidemic I | *r –* Growth rate  *K_0_* – Final size of the first sub-epidemic  *p –* Scaling of growth parameter (0 ≤ *p* ≤ 1)  *C_thr_* – Threshold value that determines when the next sub-epidemic begins  *_­_q* – Rate of decline of consecutive sub-epidemics: $K_{i}=K_{0}e^{-q(i-1)}$ |
| Sub-epidemic II | *r –* Growth rate  *K_0_* – Final size of the first sub-epidemic  *p –* Scaling of growth parameter (0 ≤ *p* ≤ 1)  *C_thr_* – Threshold value that determines when the next sub-epidemic begins *q* – Rate of decline of consecutive sub-epidemics: $K_{i}=K_{0}{(\frac{1}{i})}^{q}$ |

**Table S2.**  Average forecasting performance of each model across Situation Reports 33 – 61 for forecasting horizons of 1 – 4 weeks. The multi-model approach consists of the first four models based on calibration prediction interval coverage.

|  | PI Coverage (%) | MIS | MSE | MAE |
| --- | --- | --- | --- | --- |
| Logistic  *1 week ahead*  *2 weeks ahead*  *3 weeks ahead*  *4 weeks ahead* | 27.6  29.3  26.4  25.0 | 980.3  1047.6  1126.8  1086.3 | 1837.7  1967.3  2213.6  2083.3 | 37.2  38.0  40.8  39.5 |
| Richards  *1 week ahead*  *2 weeks ahead*  *3 weeks ahead*  *4 weeks ahead* | 41.4  43.0  44.8  47.4 | 352.1  405.1  439.4  398.1 | 920.0  1034.1  1238.2  1215.9 | 24.9  25.3  27.5  26.9 |
| Endemic Richards  *1 week ahead*  *2 weeks ahead*  *3 weeks ahead*  *4 weeks ahead* | 55.2  56.9  56.3  56.9 | 1586.9  813.3  551.8  420.3 | 1206.4  1493.2  1892.6  2158.0 | 26.2  28.5  31.3  33.2 |
| Double logistic  *1 week ahead*  *2 weeks ahead*  *3 weeks ahead*  *4 weeks ahead* | **70.4***  **74.1***  **74.1***  **74.1*** | 715.3  458.2  **305.8***  **229.7*** | 967.1  1253.9  1981.2  2939.4 | 21.6  24.1  28.2  32.1 |
| Multi-model  *1 week ahead*  *2 weeks ahead*  *3 weeks ahead*  *4 weeks ahead* | 69.0  72.4  72.4  71.6 | 419.9  **353.6***  343.4  289.5 | **708.4***  **802.5***  **1004.2***  **1027.2*** | **19.4***  **19.9***  **22.0***  **22.8*** |
| Sub-epidemic I  *1 week ahead*  *2 weeks ahead*  *3 weeks ahead*  *4 weeks ahead* | 62.1  62.1  62.1  62.9 | **317.6***  368.4  453.6  502.5 | 872.2  1089.6  1627.4  2246.6 | 21.3  24.5  28.2  31.3 |
| Sub-epidemic II  *1 week ahead*  *2 weeks ahead*  *3 weeks ahead*  *4 weeks ahead* | 58.6  60.3  59.8  61.2 | 320.3  359.8  439.9  482.1 | 883.1  1114.1  1654.8  2296.6 | 21.4  24.3  27.7  31.1 |

* Best performance with regards to the metric (column); i.e. highest prediction interval (PI) coverage, lowest mean interval score (MIS), mean squared error (MSE), and mean absolute error (MAE)

**Table S3.** Sensitivity of calibration results to the number of bootstrap realizations (*M*) for the endemic Richards model (averaged across Situation Reports within each phase, as defined by the multi-model approach).

|  | PI Coverage (%) | MIS | MSE | MAE |
| --- | --- | --- | --- | --- |
| Sit Reps 33-36: Incline  *M = 100*  *M = 300*  *M = 500* | 100.0  100.0  100.0 | 23.5  25.2  25.1 | 14.5  14.6  14.6 | 3.1  3.1  3.1 |
| Sit Reps 37-46: Oscillating I  *M = 100*  *M = 300*  *M = 500* | 89.3  91.9  92.5 | 46.2  43.0  42.4 | 114.9  114.7  114.7 | 8.5  8.5  8.5 |
| Sit Reps 47-52: Oscillating II  *M = 100*  *M = 300*  *M = 500* | 92.0  95.1  91.9 | 56.6  55.0  56.3 | 135.6  135.6  135.6 | 9.5  9.5  9.5 |
| Sit Reps 53-61: Decline  *M = 100*  *M = 300*  *M = 500* | 84.7  85.2  86.0 | 73.7  71.5  73.2 | 154.1  154.5  154.5 | 9.5  9.5  9.5 |

* Better performance is indicated by higher prediction interval (PI) coverage, and lower mean interval score (MIS), mean squared error (MSE), and mean absolute error (MAE)

**Table S4.** Sensitivity of calibration results to the number of bootstrap realizations (*M*) for the double logistic model (averaged across Situation Reports within each phase, as defined by the multi-model approach).

|  | PI Coverage (%) | MIS | MSE | MAE |
| --- | --- | --- | --- | --- |
| Sit Reps 33-36: Incline  *M = 100*  *M = 300*  *M = 500* | 92.9  100.0  100.0 | 26.8  26.2  26.2 | 22.8  22.8  22.8 | 4.2  4.2  4.2 |
| Sit Reps 37-46: Oscillating I  *M = 100*  *M = 300*  *M = 500* | 89.4  90.8  89.4 | 49.0  46.9  44.1 | 116.0  116.0  116.0 | 8.5  8.5  8.5 |
| Sit Reps 47-52: Oscillating II  *M = 100*  *M = 300*  *M = 500* | 89.5  93.5  93.5 | 59.2  58.8  56.6 | 138.0  138.0  138.0 | 9.6  9.6  9.6 |
| Sit Reps 53-61: Decline  *M = 100*  *M = 300*  *M = 500* | 86.6  88.4  88.9 | 64.8  63.9  63.6 | 130.0  130.0  130.0 | 8.5  8.5  8.5 |

* Better performance is indicated by higher prediction interval (PI) coverage, and lower mean interval score (MIS), mean squared error (MSE), and mean absolute error (MAE)


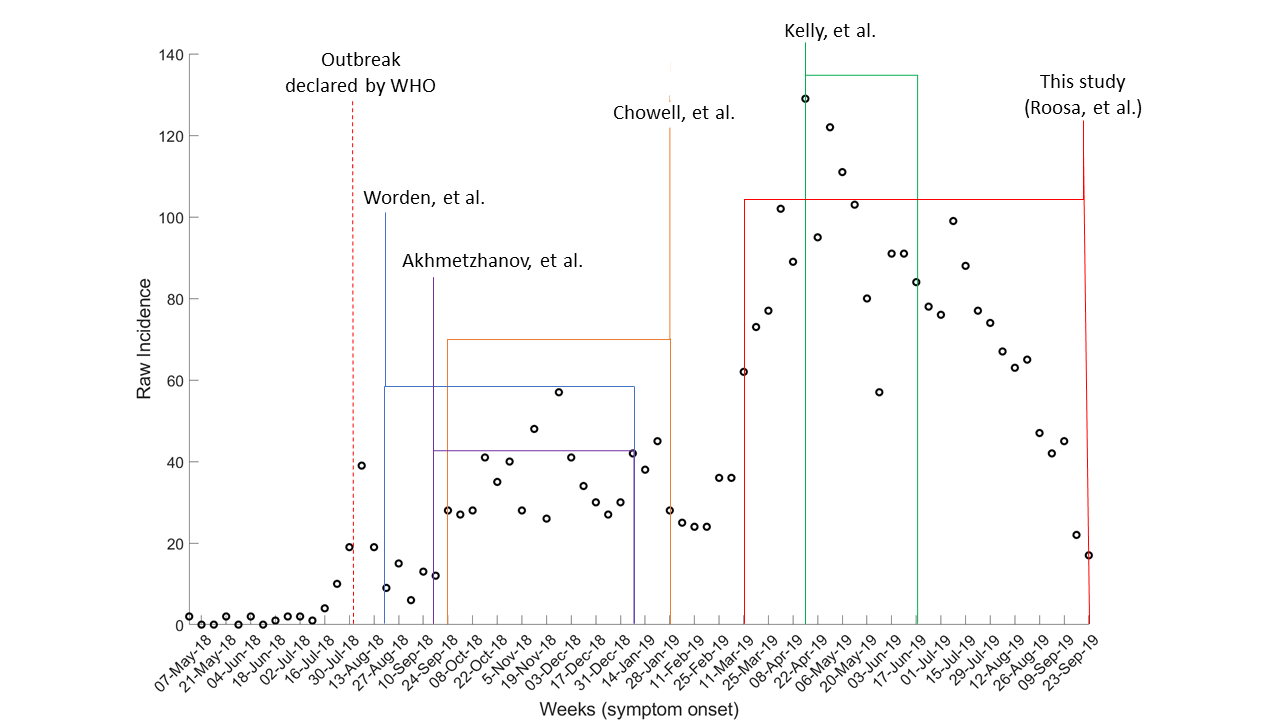
**Figure S1.** Weekly raw case incidence, as reported by the WHO, from the Ebola epidemic in the DRC. Forecasting periods from this study and previous real-time forecasting papers during the current epidemic are pictured. The outbreak was declared by the WHO on August 1, 2018.

**Figure S2.** Representative curves simulated from the double-logistic model with parameter values specified in the legend.


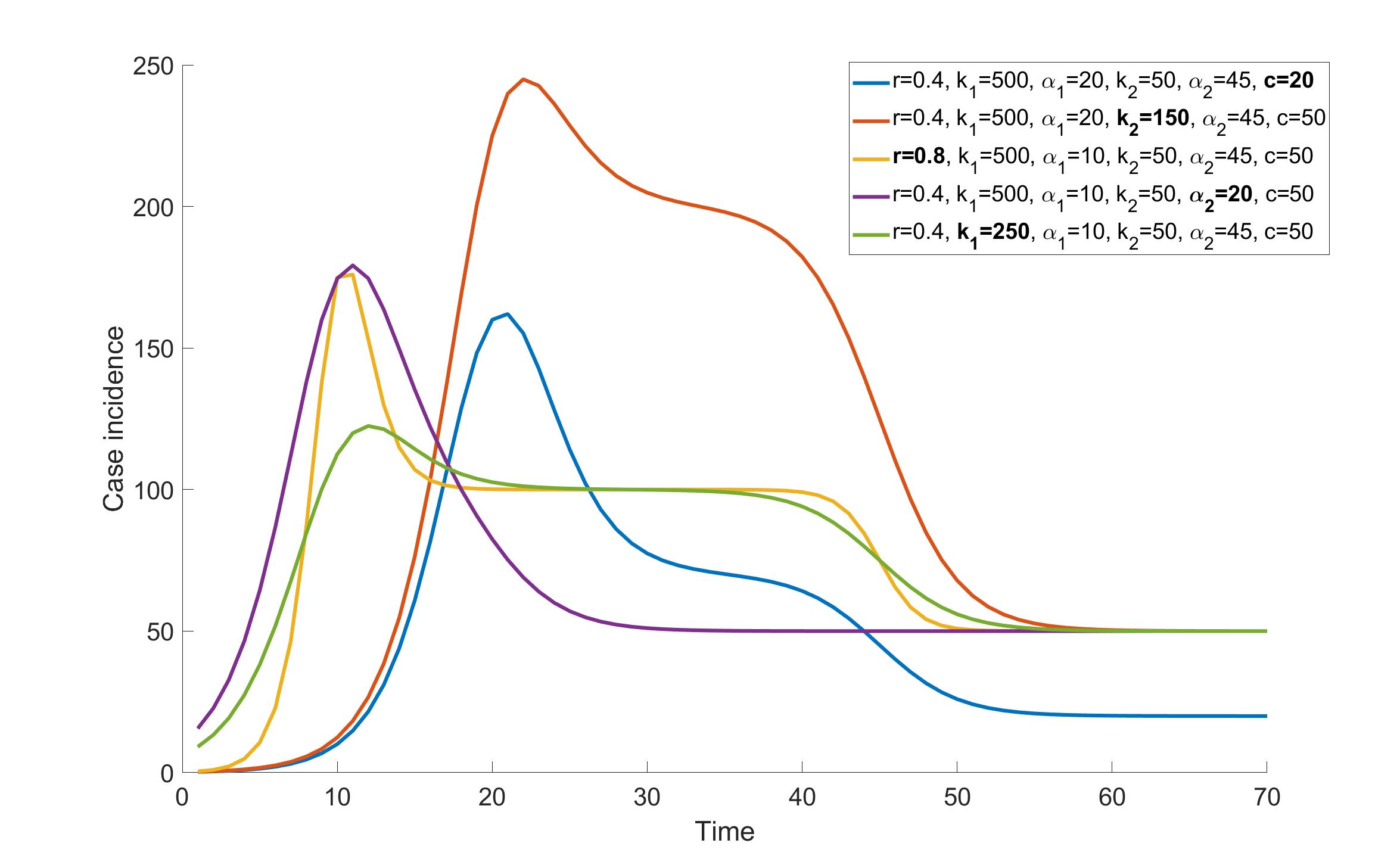


**Figure S3.** Logistic growth model – Mean model fit (solid line) and 4-week ahead forecasts with associated 95% prediction intervals (dashed lines) are presented in red with the weekly Ebola incidence data from WHO Situation Reports 33 – 61, after adjusting the data for reporting delays (black circles). The black vertical line separates the model calibration and forecasting periods.


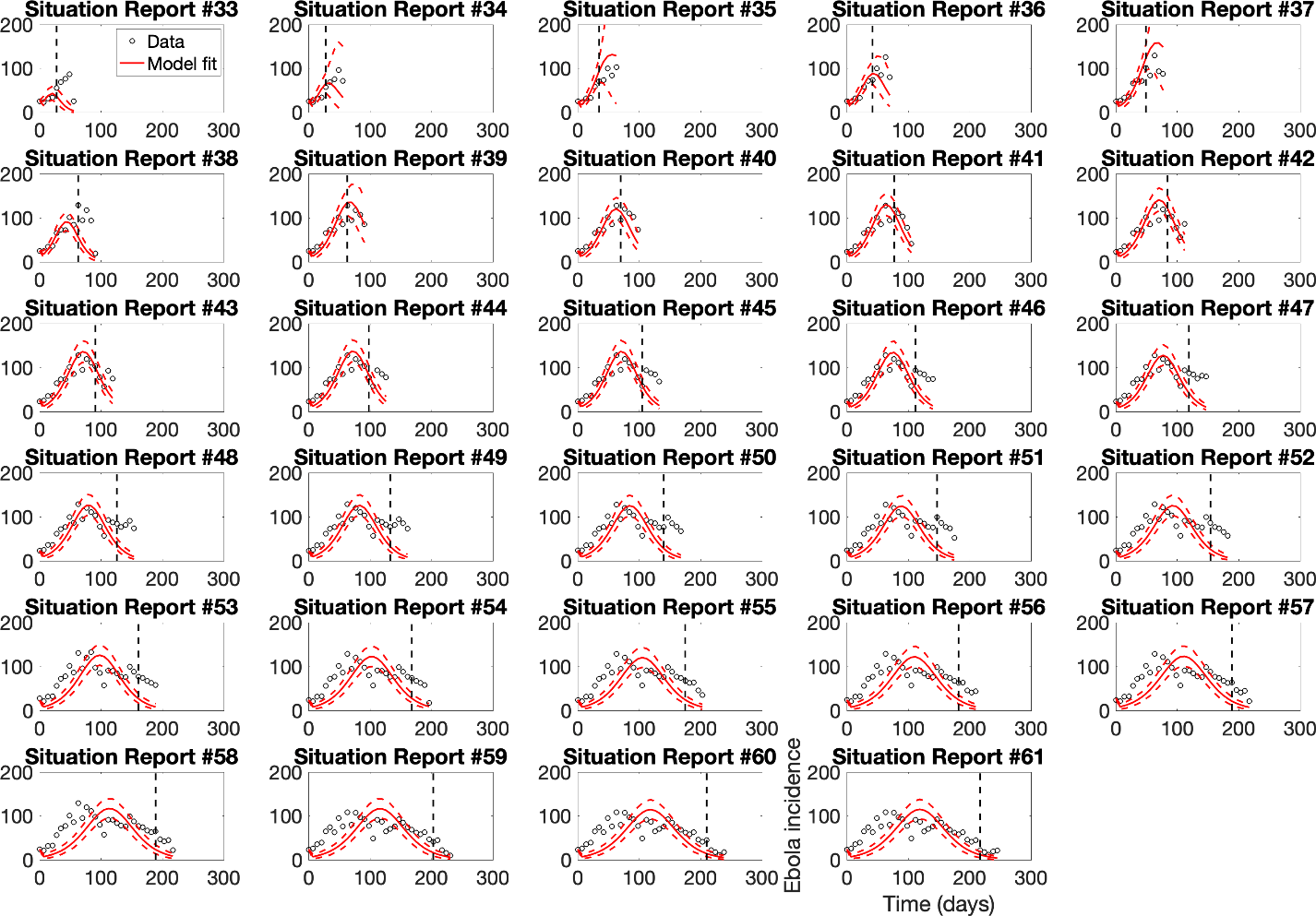


**Figure S4.** Richards model - Mean model fit (solid line) and 4-week ahead forecasts with associated 95% prediction intervals (dashed lines) are presented in red with the weekly Ebola incidence data from WHO Situation Reports 33 – 61, after adjusting the data for reporting delays (black circles). The black vertical line separates the model calibration and forecasting periods.


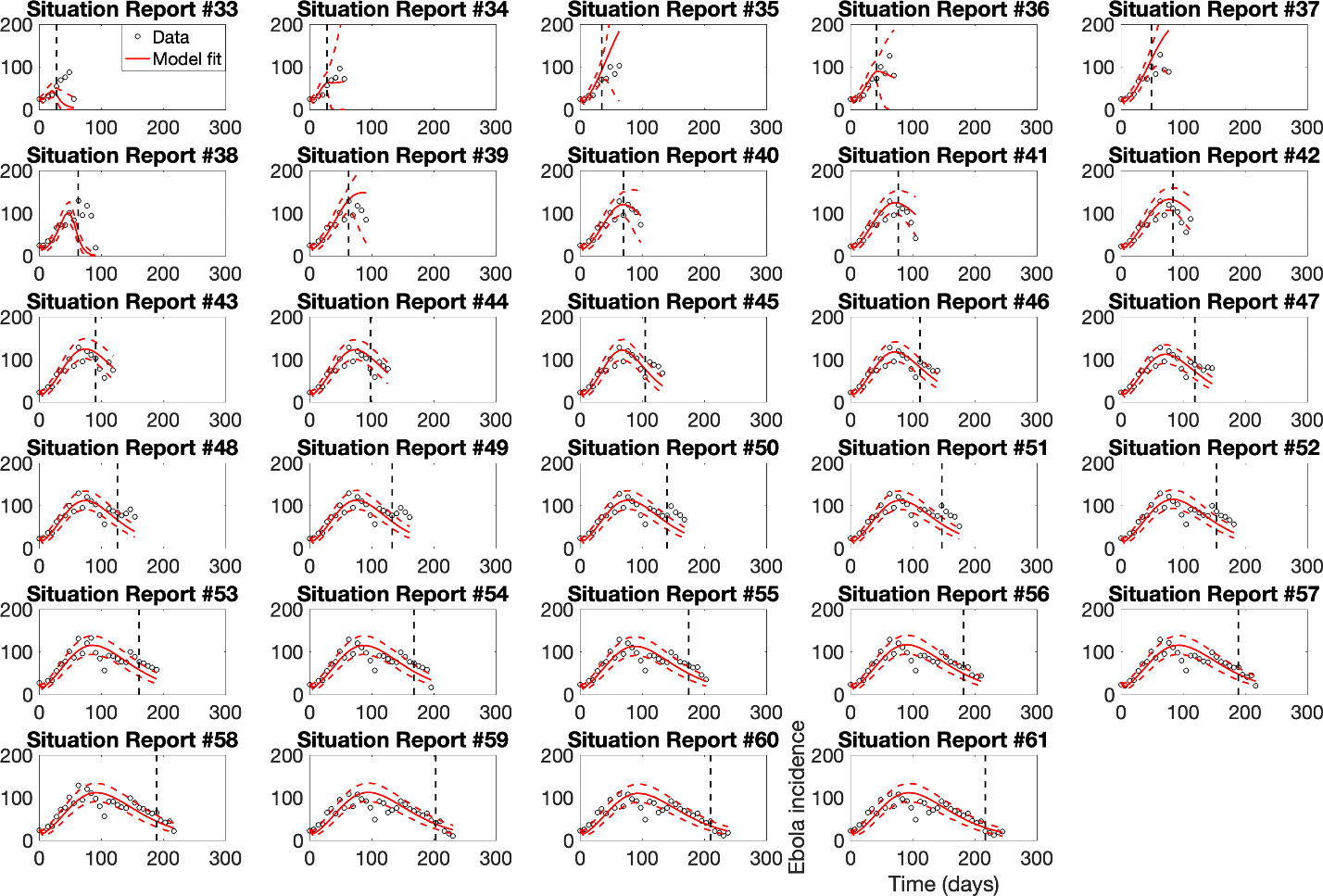


**Figure S5.** Extended Richards model - Mean model fit (solid line) and 4-week ahead forecasts with associated 95% prediction intervals (dashed lines) are presented in red with the weekly Ebola incidence data from WHO Situation Reports 33 – 61, after adjusting the data for reporting delays (black circles). The black vertical line separates the model calibration and forecasting periods.


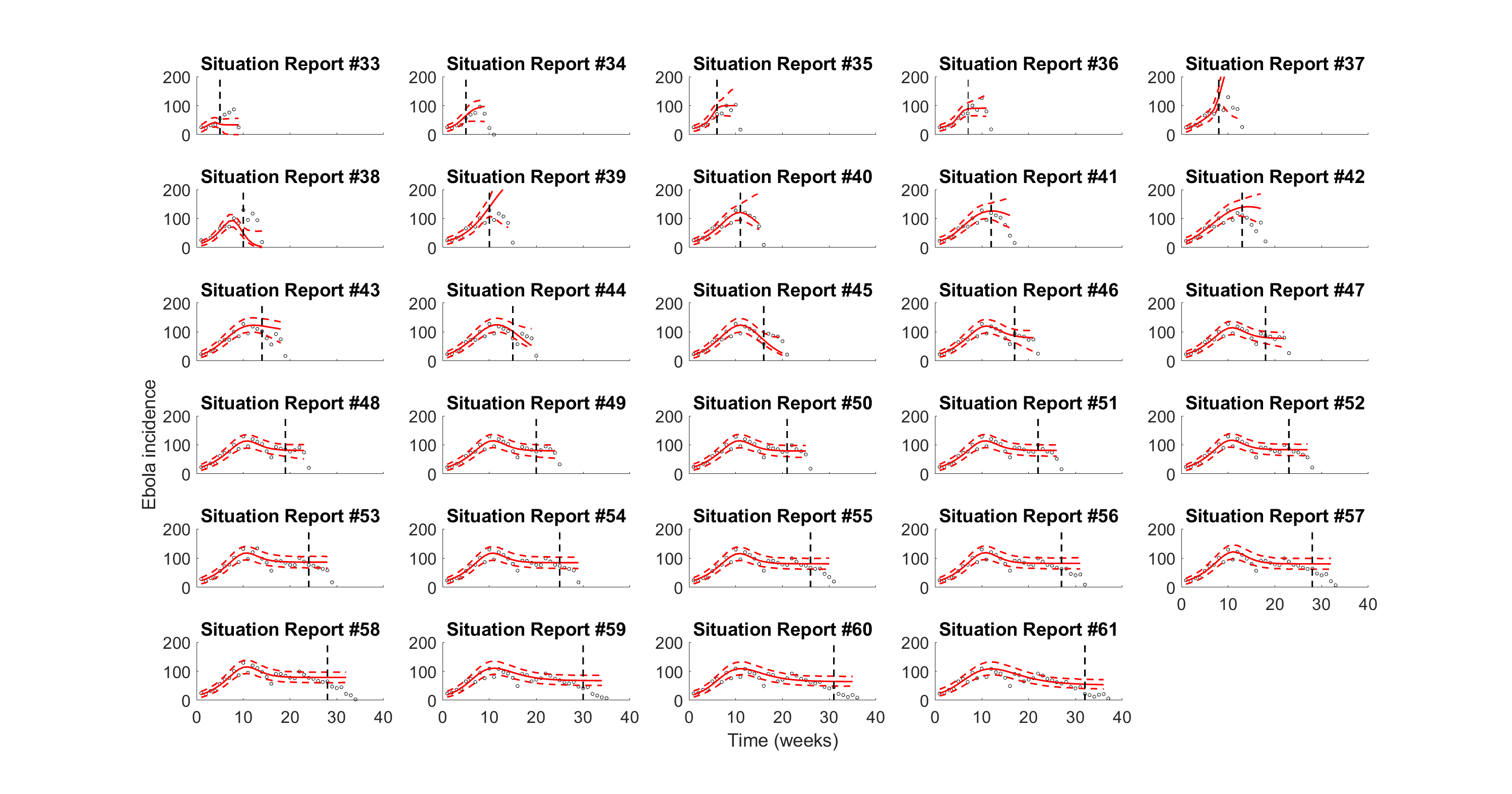


**Figure S6.** Double-logistic model - Mean model fit (solid line) and 4-week ahead forecasts with associated 95% prediction intervals (dashed lines) are presented in red with the weekly Ebola incidence data from WHO Situation Reports 35 – 61, after adjusting the data for reporting delays (black circles). The black vertical line separates the model calibration and forecasting periods.


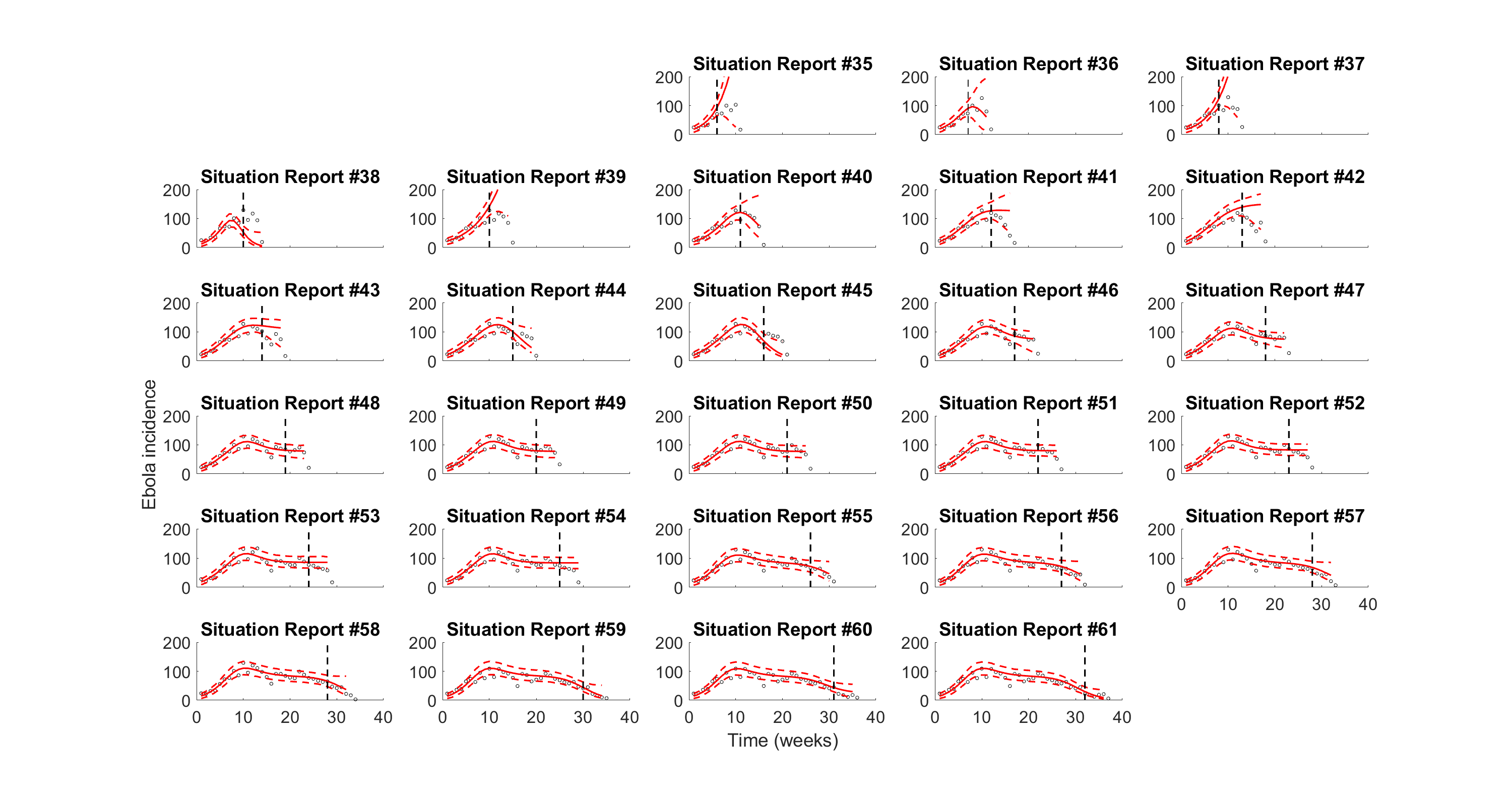


**Figure S7.** Sub-epidemic model with exponential decline function - Mean model fit (solid line) and 4-week ahead forecasts with associated 95% prediction intervals (dashed lines) are presented in red with the weekly Ebola incidence data from WHO Situation Reports 33 – 61, after adjusting the data for reporting delays (black circles). The black vertical line separates the model calibration and forecasting periods.

**
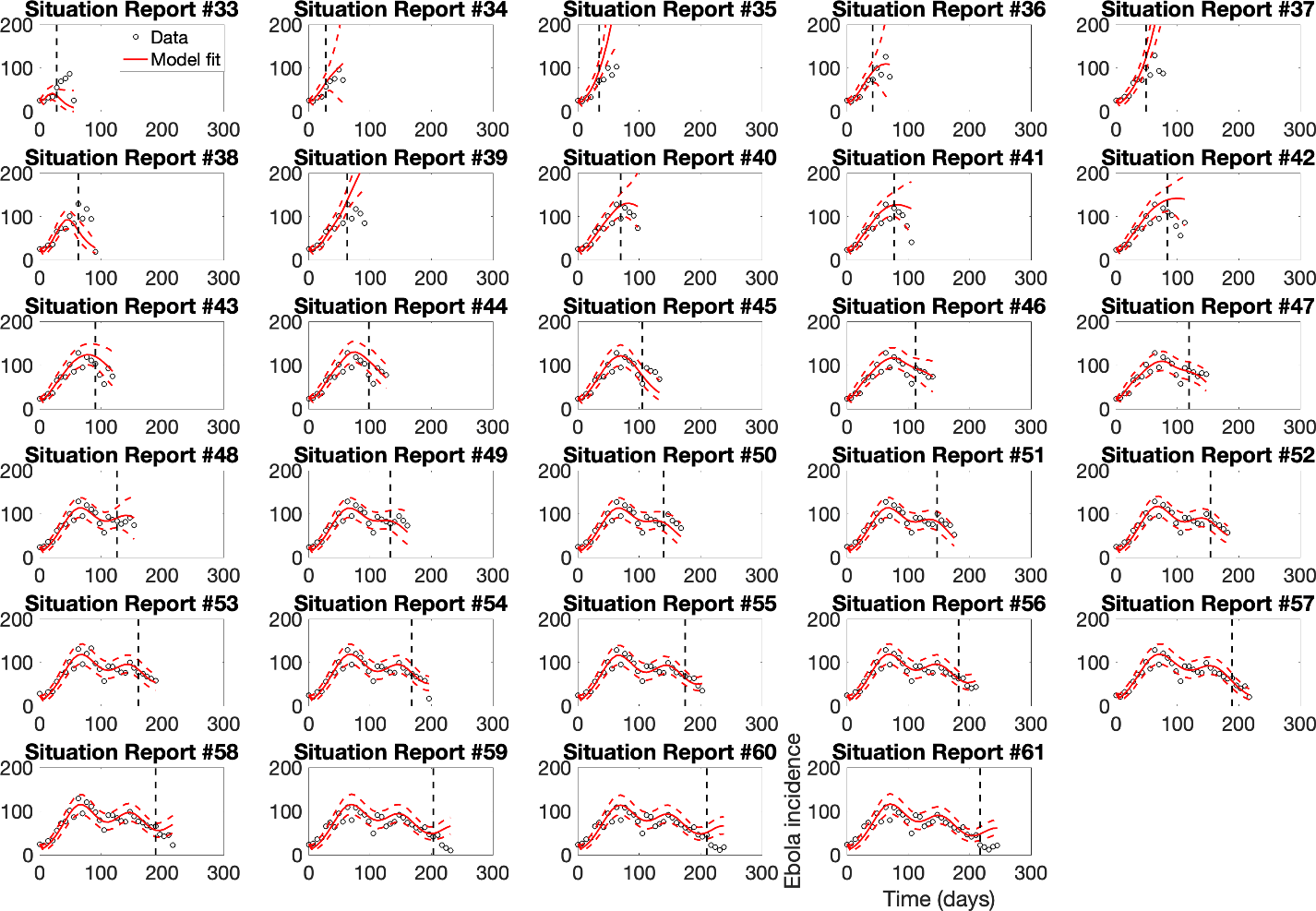
**

**Figure S8.** Sub-epidemic model with inverse decline function - Mean model fit (solid line) and 4-week ahead forecasts with associated 95% prediction intervals (dashed lines) are presented in red with the weekly Ebola incidence data from WHO Situation Reports 33 – 61, after adjusting the data for reporting delays (black circles). The black vertical line separates the model calibration and forecasting periods.


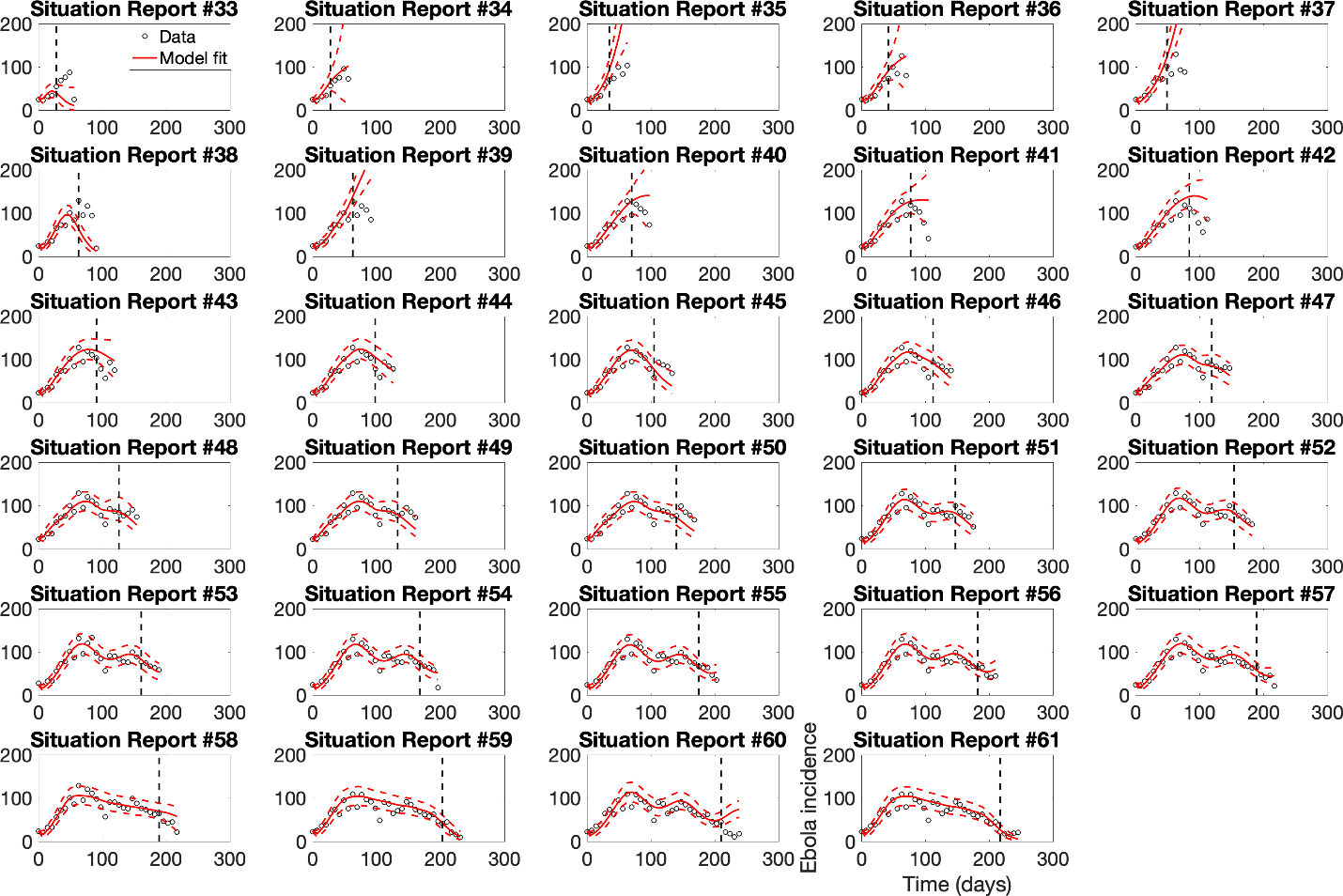


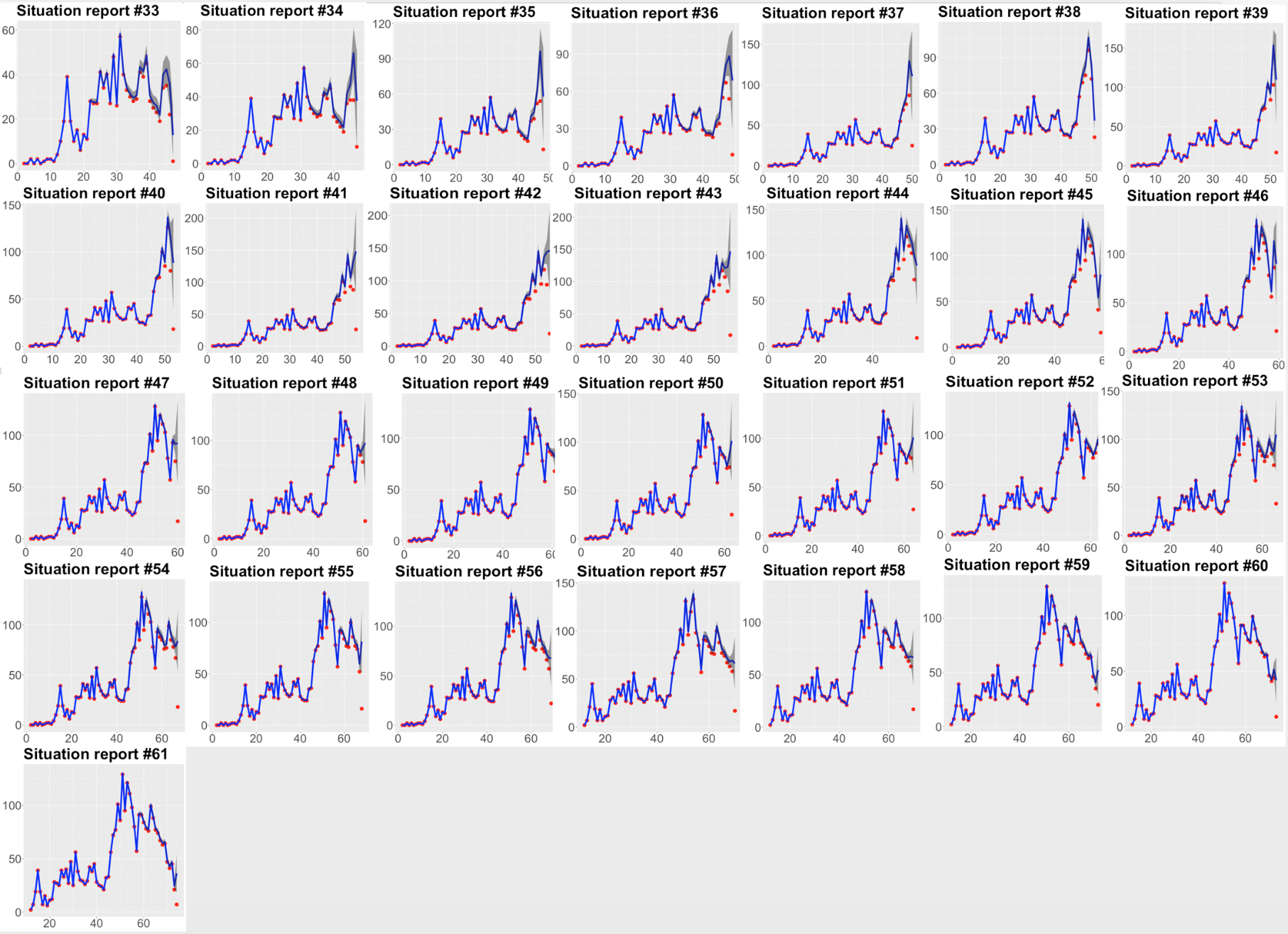
**Figure S9.** Reporting delay distribution for each WHO Situation Report (33 – 61) based on reporting delay adjustment by date of symptom onset. Reported incidence data (red points) are plotted with the mean estimate of the reporting delay adjusted incidence (solid blue line) and the associated 95% prediction intervals (dark grey shaded region).

**References**

1. Pell, B., et al., *Using phenomenological models for forecasting the 2015 Ebola challenge.* Epidemics, 2018. **22**: p. 62-70.

2. Viboud, C., et al., *The RAPIDD ebola forecasting challenge: Synthesis and lessons learnt.* Epidemics, 2018. **22**: p. 13-21.

3. Chowell, G., *Fitting dynamic models to epidemic outbreaks with quantified uncertainty: A primer for parameter uncertainty, identifiability, and forecasts.* Infectious Disease Modelling, 2017. **2**: p. 379-398.

4. Richards, F., *A flexible growth function for empirical use.* Journal of Experimental Botany, 1959. **10**(2): p. 290-301.

5. Nishiura, H., et al., *Transmission dynamics of cholera in Yemen, 2017: a real time forecasting.* Theoretical Biology & Medical Modelling, 2017. **14**(1): p. 14-14.

6. Dinh, L., et al., *Estimating the subcritical transmissibility of the Zika outbreak in the State of Florida, USA, 2016.* Theoretical Biology & Medical Modelling, 2016. **13**(1): p. 20-20.

7. Yan, P. and G. Chowell, *Quantitative Methods for Investigating Infectious Disease Outbreaks*. 2019: Springer.

8. Chowell, G., A. Tariq, and J.M. Hyman, *A novel sub-epidemic modeling framework for short-term forecasting epidemic waves.* BMC Medicine, 2019. **17**(1): p. 164-164.
